# Ethnic differences in cancer incidence in Estonia: two cross-sectional unlinked census-based cancer incidence analyses

**DOI:** 10.1186/1478-7954-7-10

**Published:** 2009-06-28

**Authors:** Katrin Lang

**Affiliations:** 1 Department of Public Health, University of Tartu, 19 Ravila St, 50411 Tartu, Estonia

## Abstract

**Background:**

Estonian and Russian ethnic groups in Estonia differ from one another in several aspects, such as historic and socio-economic background, language and culture. The aim of the current study was to examine ethnic differences in cancer incidence in Estonia, and to compare the situation before and after the profound political and economical changes in the early 1990s.

**Methods:**

Two cross-sectional unlinked census-based cancer incidence analyses were performed. Cancer incidence data were obtained from the Estonian Cancer Registry. Population denominators came from the population censuses of 1989 and 2000. Standardized cancer incidence rates were calculated for men and women for the aggregate periods 1988–1990 and 1999–2000. Differences in cancer incidence between Estonians and Russians in 1989 and 2000 were estimated for both sexes, using standardized rate ratios with 95% confidence intervals.

**Results:**

In 1988–1990, the total cancer incidence in Russian men was higher than that in Estonian men (SRR = 1.26, 95%CI = 1.19–1.34). In 1999–2000, the total cancer incidence in men showed only slightly higher estimates in Russians than in Estonians (SRR = 1.06, 95%CI = 0.99–1.32). Cancers of stomach, colon and lung had persisting higher values in Russian men in 1999–2000. In women, the differences were smaller than in men, and the total cancer incidence showed no differences relating to neither of the time periods studied. With regard to specific sites, excess of stomach cancer incidence was seen in Russian women (SRR = 1.45, 95%CI = 1.15–1.81). The ethnic differences in general decreased between the two time periods studied.

**Conclusion:**

Some of the differences in cancer rates between the Estonians and Russians in Estonia are likely to be attributable to the variation in exposure to specific etiologic factors that are causedby differences in lifestyle and habits, such as hygiene, smoking and drinking. Further research with a view to understanding these ethnic differences in cancer incidence is warranted.

## Background

Between 1918 and 1940, when Estonia was an independent country, ethnic Estonians constituted almost 90% of the population. After being annexed by the Soviet Union, and as a result of WW II, big waves of migration occurred in Estonia changing the ethnic composition considerably. In the second half of 20^th ^century, the proportion of Russian ethnic group, which has been by far the second largest over time, varied between 20 and 30% [1,2]. About one half of the Russians living in Estonia are second-generation migrants, as the distribution by country of birth showed that in 2000, 53.3% of the Russian population had been born in Estonia.

Estonian and Russian ethnic groups in Estonia differ from one another in several aspects, such as historic and socio-economic background, language and culture. During the Soviet time, issues of ethnicity in Estonia were not studied extensively. Only one study, published in Russian [3], looked at cancer rates in Estonians and Russians focusing on stomach cancer, and concluded that in 1968–1971, the Russian/Estonian ratio of age-adjusted incidence rates was 1.9–2.0 for the urban and 1.7 for the rural population. Thomson et al. [4] have detected that in the North-Eastern cities, where the population is predominantly Russian, the incidence rates for cancers of stomach, rectum, and lung were higher than the Estonian average. More recently, a study of social variation in self-rated health in Estonia [5] revealed that Russian ethnicity was responsible for 19.1% of "avoidable poor health" in men and 18.9% in women. Another study by Leinsalu et al. [6] looked at the ethnic differences in mortality and found that Russians had poorer health in general and higher mortality than Estonians for almost all selected causes of death, including cancers of stomach and lung.

The aim of the current study was to examine ethnic differences in cancer incidence in Estonia, and to compare the situation before and after the profound political and economical changes in early 1990s.

## Methods

Two cross-sectional unlinked census-based cancer incidence analyses were performed. A similar approach has been used for studying ethnic differences in mortality in Estonia after the collapse of the Soviet Union [7]. Cancer incidence data were obtained from the Estonian Cancer Registry (ECR) in the form of individual level records containing data on cancer diagnosis, year of diagnosis, patient's age in years, ethnicity, and sex. At ECR, the primary site and histological type of the tumour are routinely classified in the *International classification of diseases *(ICD) *for oncology *[8]. For the current study, these were obtained in ICD-10 [9] 4-digit code. The ten selected cancer sites for men and women are those with the highest rates in Estonia, excluding "other skin" referring to other malignant neoplasms of the skin (all other excluding melanoma), leukaemia, and cancers of the brain and nerves, as well as the aggregate of all cancer sites together excluding "other skin". Data were obtained for the years 1988–1990 and 1999–2000. The justification for selecting these years is that an attempt was made to obtain three years of cancer incidence data around both censuses under observation (those of 1989 and 2000). The Personal Data protection Act adopted in Estonia in 2003 prevents linkage of the Estonian Cancer Registry files with the death certificate database [10]. This inevitably affects reported incidence after 2000, and for that reason the year 2001 was not included in the current calculations.

Population denominators came from the population censuses of 1989 and 2000 [11]. The population denominators were available by year of age and were aggregated to form 5-year age groups.

Standardized cancer incidence rates (using European standard population, five-year age groups) were calculated for men and women for the aggregate periods 1988–1990 and 1999–2000, assuming that the ethnic distribution of the population in the census years was an unbiased estimate of the distribution for the peri-censal years. Differences in cancer incidence between Estonians and Russians in 1988–1990 and 1999–2000 were estimated for both sexes, using standardized rate ratios (SRR) with 95% confidence intervals. Calculation of confidence intervals was based on the formula presented by Breslow and Day [12]. Data analysis was performed using STATA software.

### Data quality

The 1989 population census in Estonia was carried out in the framework of all-Union censuses and on the basis of all-Union programmes [1]. No data on the coverage and/or data quality of this census are available. For the 2000 census, some quality estimates are available. For evaluating the coverage of the 2000 census and the quality of the census data, a post-enumeration sample survey was organized [13]. It covered about 1% of the population and a stratified random sample of enumeration areas was drawn. Comparison of the census data and the data collected in the post-enumeration survey showed that the under-enumeration of the census was on an average 1.2%, which proves that the coverage of the census was very high.

As for determining ethnicity, during the 1989 and 2000 censuses, it was recorded according to the statement of the person, i.e. self-determined ethnic identity [11].

At the ECR, data about ethnicity have become less complete over the more recent years and are currently about 85% complete. As for the completeness and validity of registering ethnicity at ECR, ethnicity is recorded only in about one third of the medical case notes [14]. However, according to the doctors filling in cancer notifications, if the respective pro forma about ethnicity is lacking or not completed in the medical case notes, the patient is asked about his/her ethnicity and the information is recorded on the cancer notification.

## Results

The numbers of registered cancers that were studied are presented in Table 1, relating to all cancers registered at ECR in 1988–1990 and 1999–2000. The table shows that the proportion of observations where the information about ethnicity is missing has increased considerably between these two time periods. As for the later time period, 1999–2000, the finding of missing values for ethnicity is around 14%.

**Table 1:** Distribution by ethnicity of the numbers of registered cancers studied, 1988–1990 and 1999–2000.

**Ethnicity**	**1988–1990**		**1999–2000**	
	**Number**	**%**	**Number**	**%**
**Estonian**	9559	65.2	7331	61.7
**Russian**	3831	26.1	2620	22.1
**Other**	884	6.0	284	2.4
**Missing**	378	2.6	1632	13.8
**Total**	**14652**	**100.0**	**11867**	**100.0**

The following is a comparison of the size of the relative ethnic differences in cancer incidence between Estonian and Russian men with regard to two time periods: 1988–1990 and 1999–2000 (Additional file 1). The results show that for the period 1988–1990, the total cancer incidence in Russian men was higher than that in Estonian men. As for specific sites, a number of cancer sites such as oesophagus, stomach, colon, rectum, larynx and lung had higher values in Russians when the two ethnic groups were compared. The rest of the cancer sites showed similar values for the two ethnic groups. In 1999–2000, the total cancer incidence in men showed only slightly higher estimates in Russians than in Estonians. Higher incidence in Russians persisted in cancers of stomach and lung, and appeared for cancer of pancreas. Regarding cancer of pancreas, it should be noted that the ethnic difference seen in the latter period is caused by different time trends: incidence decreased in Estonian men but not in Russian men.

The same characteristics for women are compared in Additional file 2. To summarize, in 1988–1990 the total cancer incidence in women was similar for the two ethnic groups. With regard to specific sites, some variation could be seen as cancers of oesophagus, stomach and rectum have higher rates in Russian women, and cancers of cervix uteri and bladder have lower rates in Russian women. Similarly to the earlier study period, in 1999–2000 total cancer incidence in women showed no difference between the two ethnic groups. Still, some differences occurred when specific sites were compared. Cancers of stomach, colon and pancreas had higher rates in Russian women, while cancers of breast, cervix uteri and bladder had somewhat lower rates in Russian women when compared to Estonian women. The higher rates for stomach cancer and lower rates for cancers of cervix uteri and bladder have persisted between the two points of time studied.

To summarize the changes in cancer incidence between Russians and Estonians in Estonia over time, it can be seen that the ethnic differences in cancer incidence have become smaller for nearly all cancer sites (except for some sites in women).

## Discussion

Some of the limitations of the current study should be mentioned. This study does not account for socio-economic or urban-rural differences, as these variables did not exist in the data that were used. Socio-economic conditions could have an effect on ethnic differences in cancer incidence. As stated in the methods section, the validity of recording ethnicity at ECR is rather low. Data about ethnicity are quite often based on "informal" decisions made during the cancer registration process and this may also affect the results of the current study. Also, differential reporting of ethnicity at population census and at cancer reporting (which is very unlikely) could bias the result of the current study. Another limitation could be the differential migration of the Russians to Russia, which may have caused changes in the Estonian/Russian cancer incidence rates over time.

It was seen in Table 1, that the percentage with missing ethnicity increased from 2.6% to 13.8% between the two periods studied. This could have affected the validity of the results of the current study if missing ethnicity had been predominantly on account of one of the main ethnic groups. There has been an overall decrease in the completeness of reporting of some personal identification items in the ECR over the recent years, such as marital status, ethnicity, place of birth [14]. This phenomenon makes differential reporting less likely to be on the account of one ethnic group.

Regarding the quality of cancer registration at ECR during the study period, it should be noted that the definition of primary site or registration procedures did not change. Estonian cancer data were included in *Cancer incidence in five continents*, volumes VIII and IX [15,16], and thus they are considered to be acceptable for measuring the process of cancer incidence in Estonia.

The studies on ethnicity and health in Estonia by Leinsalu et al. have shown that Russian ethnicity is the most influential factor underlying poor health [5] and that mortality differences between Estonians and Russians exist [6], with Russians having higher mortality. The current study adds a new aspect to this knowledge by showing that there are differences in cancer incidence between the two main ethnic groups in Estonia.

The fact that Russian men had a much higher total cancer incidence in 1988–1990 than Estonian men can possibly be explained by a higher incidence of lung and stomach cancer rates in Russian men in 1988–1990. High incidence of lung cancer in Russian men is explained by higher prevalence of smoking [7], especially in older Russian men reporting higher odds for ever having smoked when compared with Estonian men. When Estonia and Russia are compared as countries, lung cancer incidence in Russia is also higher than in Estonia [17]. Comparing mortality rates between ethnic groups in Estonia, Leinsalu et al. [6] have reported higher mortality in Russian men for cancers of lung and stomach. According to the same study, the differences in lung and stomach cancer mortality between Estonians and Russians decreased over time. Similar changes in cancer incidence occurred according to the current study.

Relatively high stomach cancer rates in Estonia may be explained by high prevalence of *H. Pylori *infection at around 80% [13]. According to the current study, the stomach cancer rates are higher in Russians than in Estonians. This is consistent with the findings of Rahu [3]. Still, when comparing the incidence rates for this malignancy with respective rates in Russia, it is seen that the latter are higher [17]. This points to a higher prevalence of *H. Pylori *infection in Russia that is related to poorer sanitary conditions there as compared to Estonia, at least in the past. According to Reshetnikov [18], the seroprevalence of *H. Pylori *infection in Siberian populations is 71–92%. The Russian population in Estonia may have a higher prevalence of *H. Pylori *infection inherent to their country of origin, as this infection is mainly acquired in childhood and about half of the Russian population in Estonia was born in Russia [19]. A study has detected a high prevalence of *H. Pylori *in immigrant populations in the Netherlands [20]. Studies that looked at mortality patterns of immigrants to Israel from the former Soviet Union found that the study cohort had higher SMR-s for stomach cancer relative with Israeli and German populations [21,22].

It should be noted that the potential effect of different lag-periods for *H. Pylori *and smoking in causing cancer might also explain the differences in the occurrence of stomach and lung cancers between Estonians and Russians in Estonia between 1988–1990 and 1999–2000.

The decline in pancreatic cancer incidence in Estonian men is not observed in Russian men. As smoking and alcohol consumption are risk factors for pancreatic cancer, this may be explained by differences in lifestyle, such as more extensive use of strong spirits and more prevalent daily smoking by Russian men [23] for 1990–2000. In the Netherlands Cohort Study, compared with abstention, the highest category of alcohol consumption (> or = 30 g/day of ethanol) was positively associated with pancreatic cancer risk [24]. Excessive alcohol consumption by Russian men is probably also reflected in higher rates of stomach cancer. The findings of other alcohol-related cancers, such as colorectal cancers and oesophageal cancer in this study show small differences between the two ethnic groups and are inconsistent.

## Conclusion

To conclude, the Russians in Estonia have an excess cancer rate for a number of sites, and the differences are more pronounced in men. A constant finding is the excess of stomach cancer in Russians for both sexes. Some of the differences in cancer rates between the Estonians and Russians in Estonia are likely to be attributable to the variation in exposure to specific etiologic factors that are causedby differences in lifestyle and habits, such as hygiene, smoking and drinking. Further research with a view to understanding these ethnic differences in cancer incidence is warranted.

## Competing interests

The author declares that she has no competing interests.

## Supplementary Material

Additional file 1 **Table 2. Comparison of cancer incidence rates between Estonian and Russian men in 1988–1990 and 1999–2000. Age-standardised incidence rates per 100 000 per year, all ages**. The data provided represent cancer incidence rates between Estonian and Russian men in the study periods. Click here for file

Additional file 2 **Table 3. Comparison of cancer incidence rates between Estonian and Russian women in 1988–1990 and 1999–2000. Age-standardised incidence rates per 100 000 per year, all ages**. The data provided represent cancer incidence rates between Estonian and Russian women in the study periods. Click here for file
